# Comparison of Functional Outcome of Single Versus Multiple Intra-articular Platelet-Rich Plasma Injection for Early Osteoarthritis Knee

**DOI:** 10.7759/cureus.38513

**Published:** 2023-05-03

**Authors:** Madhavan Parmanantham, Hariprasad Seenappa, Subhashish Das, Arun H Shanthappa

**Affiliations:** 1 Department of Orthopaedics, Sri Devaraj Urs Medical College, Kolar, IND; 2 Department of Pathology, Sri Devaraj Urs Medical College, Kolar, IND

**Keywords:** multiple intra-articular injections, womac, vas, early osteoarthritis knee, prp

## Abstract

Background

Osteoarthritis (OA) is a leading cause of pain and disability and has a negative impact on patients’ quality of life. Platelet-rich plasma (PRP) has emerged as a promising treatment for various orthopaedic conditions, such as tendinopathies, nonunion, and arthritis of the knee. We sought to determine whether a single intra-articular platelet-rich plasma injection has better functional and pain outcomes when compared with multiple (two) articular platelet-rich plasma injections given in the early stages of OA of the knee, measured using the Western Ontario and McMaster Universities Arthritis Index (WOMAC) and the visual analogue scale for pain (VAS) at the sixth week, third month, and sixth month.

Materials and methods

The prospective observational study was conducted among patients diagnosed with early OA who presented to the Department of Orthopaedics, R. L. Jalappa Hospital and Research Centre, Kolar, Karnataka, India, between January 2020 and June 2021. A total of 64 patients were divided into: (i) S-PRP group (34 patients), which received a single PRP injection, and (ii) M-PRP group (30 patients), which received multiple (two) PRP injections, one on presentation and the second in the thirdmonth. VAS and WOMAC scores to assess functional outcomes were used at the first visit before the intervention and at the sixth week, third month, and sixth month.

Results

The average age of the patients was 55.26 years in the S-PRP group and 51.13 years in the M-PRP group. Both genders were equal among study participants in the M-PRP group, but 79.4% were females in the S-PRP group. In the S-PRP group, 74% had grade II OA and 26% had grade I OA. In the M-PRP group, 60% had grade II OA and the remaining 40% had grade I OA. The decreasing trend of pain and functional limitation, which was measured by VAS and WOMAC, respectively, was observed in both groups at pre-injection, sixth week, third month, and sixth month. These differences were statistically significant. The mean difference in VAS score between the pre-injection period and at six months was 4 in the S-PRP group, whereas it was 5.77 in the M-PRP group, and this was statistically significant (p-value = 0.001). Thus, multiple PRP injections have a greater response to pain reduction when compared to single PRP injections, according to the VAS score. According to the WOMAC score, there is no statistically significant difference in the treatment response with PRP injection between the S-PRP and M-PRP groups at any follow-up period.

Conclusion

According to the VAS score, single PRP injections have a lower pain score than multiple PRP injections until three months of follow-up, while at six months, single PRP injections have no better effect than multiple PRP injections. But multiple PRP injections have a higher reduction in the intensity of pain when compared to single PRP injections during the follow-up period. According to the WOMAC score, there is no statistically significant difference in the treatment response with PRP injection between S-PRP and M-PRP groups.

## Introduction

Osteoarthritis (OA) is a multifactorial degenerative illness characterized by articular cartilage loss, bone enlargement at the borders, subchondral sclerosis, and a variety of biochemical as well as morphological changes in the synovial membrane as well as the joint capsule [1].

OA is the second most widespread rheumatologic illness and the most common joint illness in India, with an occurrence rate of 22-39%. Women are more likely than men to have OA, although the prevalence rises drastically with age. Nearly half of all women above 65 years have symptoms, and 70% of those above 65 years show radiographic evidence. Knee OA is a general cause of mobility loss, especially in women. The 10th greatest cause of nonfatal burden was projected to be OA [2,3]. However, more recent research has recognized and labelled an overabundance of additional factors contributing to knee OA pathogenesis [4].

Early knee OA is defined as the presence of at least two episodes of knee pain lasting 10 or more days in the last year, a Kellgren-Lawrence grade of 0, I, or II, and either arthroscopic confirmation of cartilage lesions or magnetic resonance imaging findings demonstrating articular cartilage degeneration and/or meniscal degeneration and/or subchondral bone marrow lesions [5]. OA of the knee is a major issue that ageing adults face, and to alleviate the pain and morbidity associated with OA pain, physicians and orthopaedics around the world have tried a variety of non-surgical treatment modalities ranging from oral chondro-protectives, intra-articular steroids, and viscosupplements. Platelet-rich plasma (PRP) is becoming a viable treatment option for a variety of orthopaedic disorders, including tendinopathies, non-union, and knee arthritis. The effectiveness of PRP in treating sports injuries in several high-profile athletes has helped fuel the hype around PRP therapy, resulting in an increase in PRP use for treating OA knees over the last seven years [6].

PRP is an autologous blood component with a high concentration of platelets that is used to treat bone, tendon, and ligament injuries in orthopaedic and sports medicine practices [7,8]. In addition, PRP injections can be used to treat cartilage damage and OA [9,10]. Normal joint metabolism is altered by OA, supporting increased catabolism and diminished anabolism. Platelet alpha-granules comprise and discharge a variety of growth factors, such as hepatocyte growth factor, vascular endothelial growth factor, platelet-derived growth factor, and transforming growth factor-b (TGF-b), all of which may affect the altered joint milieu in OA. PRP affects joint homeostasis on multiple levels [11,12]. In cartilage, it reduces catabolism and improves anabolism, which in turn promotes chondral remodelling. A high content of collagen II and prostaglandin (PG) synthesis has been found in the research done by Akeda et al. and Pereira et al [13,14]. Raising chondrocyte proliferation and the production of matrix molecules have also been found in the study [15].

Regardless of hopeful preclinical outcomes and widespread clinical curiosity in orthopaedics as well as sports medicine, there are still many unsolved concerns about PRP's therapeutic applicability and efficacy. There is ambiguity regarding the number and frequency of injections required for optimal efficiency, as well as the best treatment for various stages of gonarthrosis (from cartilage injury to advanced OA) [16]. The present study aims to determine whether a single intra-articular PRP injection versus multiple intra-articular PRP injections administered in the early stages of knee OA have a better functional outcome, as measured using the Western Ontario and McMaster Universities Osteoarthritis Index (WOMAC), and pain outcome, as assessed using a visual analogue scale for pain (VAS), at six weeks, three months, and six months.

## Materials and methods

The prospective observational study was conducted among patients with early stages of osteoarthritis who presented between January 2020 and June 2021 to the Department of Orthopaedics, R. L. Jalappa Hospital and Research Centre attached to Sri Devaraj Urs Medical College affiliated to Sri Devaraj Urs Academy of Higher Education and Research Tamaka, Kolar, Karnataka, India. The Institutional Ethics Committee of Sri Devaraj Urs Medical College approved the study (approval number: DMC/KLR/IEC/614/2021-22).

Inclusion and exclusion criteria

Inclusion criteria were: (i) clinical signs of OA, (ii) chronic knee pain for more than four months, (iii) radiologically documented grade I-II knee osteoarthritis (Kellgren - Lawrence), and (iv) patient age between 40 and 60 years, Exclusion criteria were: (i) previous lower extremity surgery, (ii) rheumatic diseases and other inflammatory diseases in the knee, (iii) severe cardiovascular disease, (iv) haematological diseases, (v) infections in the knee, (vi) patients with haemoglobin values less than 11 g/dL, and (vii) platelet values less than 150,000/mm^3^.

Sample size calculation

\(n=2Sp^{2} [Z_{1-\α /2} + Z_{1-\β }]^{2}/{u^{2}d}

Sp^{2} = S_{1}^{2}+S_{2}^{2}/2S_{1}^{2}\)

S_1_^2^ - standard deviation in the first group; S_2_^2^ - standard deviation in the second group; μ^2^d - mean difference between the samples; α - Significance level; 1-β - Power

S_1_^2^ - 10.8

S_2_^2^ - 6.3

μ^2^d - 9.4

α - 1%

Power - 90%

The sample difference was based on the difference in visual analogue score reported between single intra-articular PRP injection and multiple intra-articular PRP injections for early OA patients at six months, as mentioned in the randomized controlled trial conducted by Gormeli et al. in their study in Turkey in 2015 [16].

The required sample size was calculated as 27 per group. With an expected dropout rate of 10% in follow-up, the estimated sample size was 30 per group. So, the minimum sample in each group was calculated as 30 or a minimum total of 60 samples.

Randomization

The samples of 64 patients who satisfied the inclusion and exclusion criteria were randomized into two groups by an online random number generator. Subjects were evaluated through proforma, and informed consent was obtained. Patients were divided into: (i) S-PRP group, in which participants would receive a single intra-articular PRP injection, and (ii) M-PRP group, where patients would receive multiple (two) intra-articular PRP injections, one on presentation and one in the third month. All the subjects were interrogated and inspected to warrant that the criteria were fulfilled. This trial was a single-blind study in which the participants were not informed of the intervention they obtained. The principal investigator and the treating staff were not blinded. Pre-intervention knee pain and functional status were assessed by VAS and WOMAC, repeated immediately after six weeks of intervention.

Data collection procedure

The selected patients, after giving their consent correctly, were subjected to a thorough history and physical examination. Patients were graded according to the Kellgren-Lawrence classification grades I-II for tibiofemoral joint degeneration. They were then subjected to X-ray knee standing anteroposterior (AP) view and 30-degree flexion lateral view.

Intervention

About 150 ml of whole blood will be collected in a double blood bag containing 63 ml of citrate phosphate dextrose adenine (CPDA) and stored at room temperature (20-24 °C) until parting, which was done within one hour of collection. Primarily, blood was centrifuged by means of a light spin at 2630 revolutions per minute (RPM) for three minutes and at 1500 RPM for another 15 minutes to sediment the RBCs and WBCs. Subsequently, the blood bag was taken out, and the supernatant PRP was transported in the transfer bag under laminar airflow. This was followed by the sealing of the primary bag with tube sealer. After an hour of resting, platelets were re-suspended within the plasma. A minimum of 15 ml of PRP was collected and succumbed to diagnostic evaluation regarding the platelet count, sterility, and relevant serological investigations before being injected into the joint.

The skin was sterilely dressed, and each injection was given by an unblinded physician using the superolateral approach with a 22-G needle; 2 ml of PRP was administered in the right, left, or both knees. The knee was immobilised for 10 minutes after the injection, and a sterile compression dressing was applied.

Data analysis

The collected data were entered in Microsoft Excel (Microsoft Corporation, Redmond, Washington, United States) and analysed using IBM SPSS Statistics for Windows, Version 23.0 (Released 2015; IBM Corp., Armonk, New York, United States). The data was described in descriptive statistics as frequency analysis, percentage analysis was used for discrete variables. Mean and standard deviation were used for continuous variables. Continuous variables in the two groups were compared for statistically significant differences using Independent T-test. Paired T-test was applied to compare the efficacy of a single PRP injection versus multiple PRP injections before and after intervention. The probability value of 0.05 was considered significant in all of the above statistical analyses.

## Results

In the present study, 64 people (34 in the single-dose PRP arm (S-PRP group) and 30 in the multiple-dose PRP arm (M-PRP group)) were included and analyzed for the results. The socio-demographic profile is shown in Table 1. The mean age of the study participants was 55.26 years in the S-PRP group and 51.13 years in the M-PRP group. Both genders were equal among study participants in the M-PRP group, but 79.4% were females in the S-PRP group. Regarding body mass index (BMI), the mean BMI was 27.94 in the S-PRP group and 27.37 in the M-PRP group. Overall, 70.31% of the study participants had right knee complaints, 15.63% had left knee complaints, and 14.06% had both right and left knee complaints. Moreover, 67.6% of the patients in the S-PRP group and 73.3% of the patients in the M-PRP group had complaints in the right knee, which is comparable. In the S-PRP group, 74% had grade II OA and 26% had grade I OA. In the M-PRP group, 60% had grade II OA and the remaining 40% had grade I OA. Further, 23.5% of the patients in the S-PRP group and 33.3% of those in the M-PRP group had diabetes; 20.6% of those in the S-PRP group and 20% of those in the M-PRP group had hypertension. In our study, 11.7% of the patients in the S-PRP group reported pain, whereas 16.7% of those in the M-PRP group reported pain. Swelling was reported in 5.8% of the patients in the M-PRP group and in 14.4% of those in the M-PRP group after treatment as a complication.

**Table 1:** Socio-demographic profile of study participants. S-PRP group: Participants receiving a single intra-articular platelet-rich plasma injection; M-PRP group: Participants receiving two intra-articular platelet-rich plasma injections

Parameters		S-PRP group (n=34)	M-PRP group (n=30)
Age (years)		55.26 ± 4.8	51.13 ± 7.41
Gender	Male	27 (79.4%)	15 (20.6%)
	Female	7 (50%)	15 (50%)
BMI		27.94 ± 1.74	27.37 ± 1.54
Affected knee	Both	4 (11.8%)	5 (16.7%)
	Left	7 (20.6%)	3 (10%)
	Right	23 (67.6%)	22 (73.3%)
Osteoarthritis	Grade 1	9 (26%)	12 (40%)
	Grade 2	25 (74%)	18 (60%)
Co-morbidity	Type II diabetes mellitus	8 (23.5%)	10 (33.3%)
	Hypertension	7 (20.6%)	6 (20%)
Complications	Injection site pain	4 (11.7%)	5 (16.7%)
	Swelling	2 (5.8%)	4 (13.4%)

Pain outcome

The S-PRP group had a mean VAS score of 6.68 before injection, which reduced to 2.68 at the end of six months, whereas the mean VAS score was 5.29 at six weeks and 3.85 at three months. Before injection, the mean VAS score in the M-PRP group was 7.83, which was reduced to 2.07 after injection at the end of six months, whereas it was 6.03 at six weeks and 4.7 at three months.

**Figure 1:**
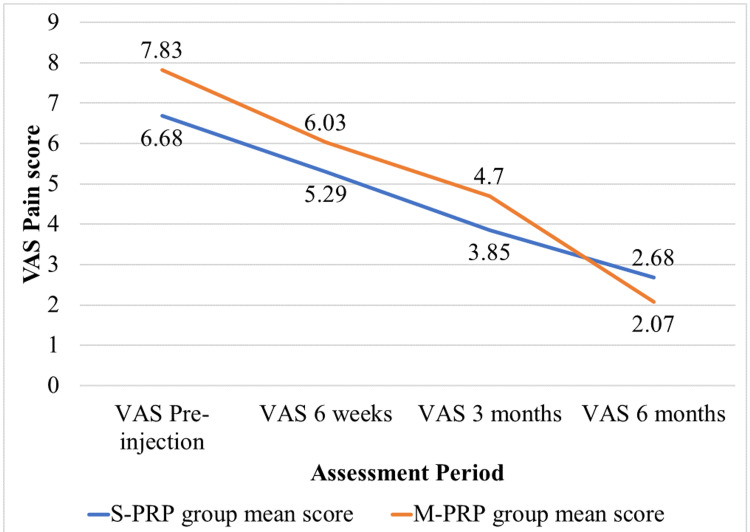
Declining trend of VAS score among the study participants (n=64; S-PRP group: 34 and M-PRP group: 30) S-PRP group: Participants receiving a single intra-articular platelet-rich plasma injection; M-PRP group: Participants receiving two intra-articular platelet-rich plasma injections VAS: visual analogue scale for pain

We have plotted the declining trend of VAS scores among the study participants in Figure 1. The decreasing trend of pain, which was measured by the VAS, was observed in both groups at pre-injection and at the sixth week, third month, and sixth month follow-ups after the injection. These differences were statistically significant.

The mean difference in VAS score between the pre-injection period and at six months was 4 in the S-PRP group, whereas it was 5.77 in the M-PRP group, and this difference in the mean is statistically significant (P value = 0.001). Thus, multiple PRP injections have a greater response to pain reduction when compared to single PRP injections, according to the VAS score. 

The mean VAS score before injection in the S-PRP group was 6.68, and in the M-PRP group, it was 7.83. This difference in means in both groups is statistically significant (p = 0.0001). In the six-week follow-up, the mean VAS scores were 5.29 and 6.03 in the S-PRP and M-PRP groups, respectively. This difference in means in both groups is statistically significant (p = 0.0002). Similarly, in the third-month follow-up, the mean scores were 3.85 and 4.7 in the S-PRP and M-PRP groups, respectively. This difference in means in both groups is statistically significant (p = 0.0001). The mean VAS score at six months was 2.07 and 2.68 in the S-PRP and M-PRP groups, respectively. This difference in means in both groups is not statistically significant (p = 0.088). Thus, according to the VAS score, single PRP injections have a lower pain score than multiple PRP injections until three months of follow-up, while at six months, single PRP injections have no better effect than multiple PRP injections. We have mentioned the comparison of VAS scores between S-PRP group participants versus M-PRP group participants in Table 2.

**Table 2:** Comparison of VAS score between single PRP injection versus multiple PRP injection among the study participants. S-PRP group: Participants receiving a single intra-articular platelet-rich plasma injection; M-PRP group: Participants receiving two intra-articular platelet-rich plasma injections VAS: visual analogue scale for pain; PRP: platelet-rich plasma

VAS Assessment Period	Group	Number	Mean	Mean Difference	P-Value
Pre-injection	S-PRP	34	6.68	-1.157	0.0001
	M-PRP	30	7.83		
6 weeks	S-PRP	34	5.29	-0.74	0.0002
	M-PRP	30	6.03		
3 months	S-PRP	34	3.85	-0.85	0.0001
	M-PRP	30	4.7		
6 months	S-PRP	34	2.68	-0.61	0.088
	M-PRP	30	2.07		

Functional outcome

The mean WOMAC score of the S-PRP group in the pre-injection period was 55.53, which was reduced to 26.8 at six months, whereas it was 45.26 at six weeks and 35.15 at three months. The mean WOMAC score of the M-PRP group in the pre-injection period was 54.87, which was reduced to 24.67 at six months, whereas it was 45.33 at six weeks and 36.1 at three months. The decreasing trend of functional restriction, measured by the WOMAC, was observed before and after intervention in both groups at pre-injection, sixth week, third month, and sixth month. These differences were statistically significant. 

**Figure 2:**
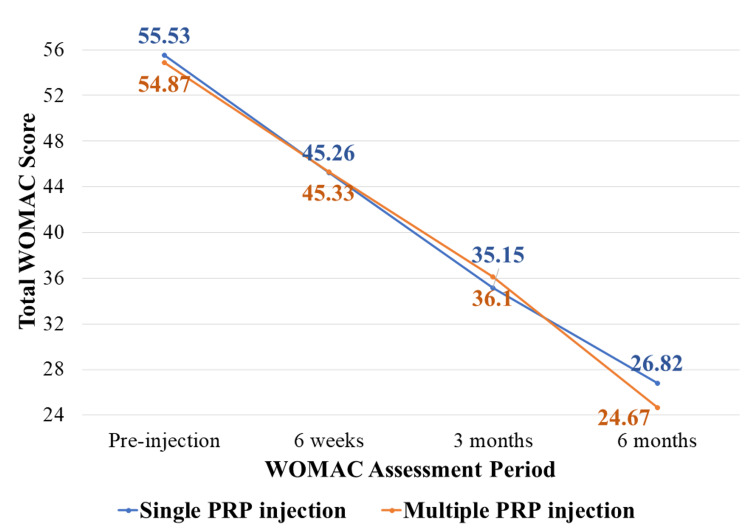
Declining trend of WOMAC scores among the study participants (n = 64; S-PRP group: 34 and M-PRP group: 30) S-PRP group: Participants receiving a single intra-articular platelet-rich plasma injection; M-PRP group: Participants receiving two intra-articular platelet-rich plasma injections WOMAC: Western Ontario and McMaster Universities Osteoarthritis Index

We have plotted the declining trend of WOMAC score among the study participants in Figure 2. The mean difference in WOMAC scale score between the pre-injection period and at six months was 28.71 in the S-PRP group, whereas it was 30.2 in the M-PRP group, and this difference in the mean is statistically significant (P value= 0.001). Thus, multiple PRP injections have a greater response in reducing functional limitations when compared to single PRP injections, according to the WOMAC score.

The mean WOMAC score before injection in the S-PRP group was 55.53, and in the M-PRP group, it was 54.87. This difference in means in both groups is not statistically significant (p = 0.286). The mean WOMAC score in the S-PRP group was 45.26 and the M-PRP group was 45.33 in the sixth week of follow-up. This difference in means in both groups is not statistically significant (p = 0.920). Similarly, the mean score in the S-PRP group was 35.15 and the M-PRP group was 36.10 in the third month of follow-up. This difference in means in both groups is not statistically significant (p = 0.118). The mean WOMAC score at six months in the S-PRP group was 26.82, and in the M-PRP group, it was 24.67. This difference in means in both groups is not statistically significant (p = 0.208). Thus, according to the WOMAC score, there is no statistically significant difference in the treatment response with PRP injection between the S-PRP and M-PRP groups. We have mentioned the comparison of WOMAC score between single PRP injection versus multiple PRP injections among the study participants in Table 3.

**Table 3:** Association between S-PRP and M-PRP groups according to WOMAC score S-PRP group: Participants receiving a single intra-articular platelet-rich plasma injection; M-PRP group: Participants receiving two intra-articular platelet-rich plasma injections WOMAC: Western Ontario and McMaster Universities Osteoarthritis Index

WOMAC Assessment Period	Group	N	Mean	Mean Difference	P-Value
Pre-injection	S-PRP	34	55.53	0.663	0.286
	M-PRP	30	54.87		
6 weeks	S-PRP	34	45.26	- 0.069	0.920
	M-PRP	30	45.33		
3 months	S-PRP	34	35.15	- 0.953	0.118
	M-PRP	30	36.10		
6 months	S-PRP	34	26.82	2.157	0.208
	M-PRP	30	24.67		

## Discussion

PRP has recently gained popularity as a treatment option for early knee OA. This study included patients in the early stages of OA. A PubMed search in November 2021 using the terms “osteoarthritis of the knee” and “platelet-rich plasma” yielded only a few studies of varying quality that used numerous injections of intra-articular PRP to treat knee OA. Some were brief pilot experiments with no controls, whereas other studies used multiple injections. Furthermore, the current literature lacks consistency in study methods, platelet separation methodologies, and outcome measurements. As a result, the evidence for PRP and autologous blood concentrate as a treatment strategy for most orthopaedic disorders, including knee OA, is mixed.

A decreasing trend of pain, which was measured by the VAS, was observed before and after intervention in both groups at pre-injection, sixth week, third month, and sixth month in the current study. According to the VAS score, single PRP injections have a lower pain score than multiple PRP injections until three months of follow-up, while at six months, single PRP injections have no better effect than multiple PRP injections. But multiple PRP injections have a higher reduction in the intensity of pain when compared to single PRP injections during the follow-up period, according to the VAS pain score. The reason could be that two injections could have reduced the intensity of the pain.

A decreasing trend of functional restriction, which was measured by the WOMAC scale, was observed before and after intervention in both groups at pre-injection, sixth week, third month, and sixth month. The lower WOMAC score is due to both therapies improving functional status by reducing pain. According to the WOMAC score, there is no statistically significant difference in the treatment response with PRP injection between the S-PRP and M-PRP groups.

Chouhan et al. (2019) conducted a controlled laboratory study in guinea pigs that sheds light on the histological basis underlying the superiority of numerous PRP injections. According to the researchers, single PRP and multiple PRP injections had the same anti-inflammatory effects on the synovium in the short term. However, this effect only lasts when multiple injections are administered, as multiple PRP injections only provide short-term chondroprotection. This effect is not produced by a single injection of PRP [17]. According to Patel et al. (2013), who conducted a randomized controlled trial in India, there was no significant change in the WOMAC scores in groups treated with either one or two injections of PRP at six months. Single or double PRP injections in the knees helped alleviate pain better than saline injections among patients with mild to moderate OA [18].

In 2019, Mentia et al. conducted a randomized controlled trial that included a single application (18 patients) and a triple application (17 patients). They found that both treatments significantly reduced VAS and total WOMAC scores [19]. When the results were compared between groups, the triple application showed a greater improvement in VAS and overall WOMAC ratings.

Tavassoli et al. (2019) published a randomized controlled trial with 95 patients in Iran and discovered that two injections of PRP were more effective in reducing pain and increasing functional status at each follow-up than a single injection [20]. According to a systematic review published in 2013 by Khoshbin et al., multiple successive intra-articular PRP injections may have favourable results in functional status among adult patients with mild to moderate knee OA at six months [21].

According to Gormeli et al. (2017), compared with a single injection of PRP or hyaluronic acid, knees treated with three injections of PRP had significantly better pain and functional scores [16]. In a randomized controlled trial comparing one with three PRP injections, Kavadar et al. (2015) reported that two and three PRP injections were significantly superior to a single injection in terms of pain and functional scores at six months [22].

Limitations

This study has several limitations. Since pain in daily activities is the most pressing problem in OA, this study’s evaluation of the disease was limited to clinical parameters using the WOMAC and VAS scoring systems. Additionally, double or triple blinding should be done in future studies to improve the accuracy and objectivity of clinical outcomes. We believe that long-term follow-up screenings lasting up to two years will provide a more accurate picture of clinical outcomes. Furthermore, very few patients with bilateral knee OA were included in the study, and the patients were randomly assigned instead of the knees. More research is needed to confirm the findings and their longevity, understand the mechanism of action of PRP, and determine whether there is only a temporary improvement or if PRP plays a more important role through its disease-modifying properties. The standardization of PRP dosing regimens is also an important research component. Moreover, post-injection rehabilitation protocols and an adequate and consistent description of the injection techniques may also improve clinical outcomes.

## Conclusions

The decreasing trend of pain and functional limitation, which was measured by the VAS and the WOMAC, was observed before and after intervention in both groups at pre-injection, 6th week, 3rd month, and 6th month, respectively, in the current study. According to the VAS scale score, single PRP injections have a lower pain score than multiple PRP injections until three months of follow-up, while at six months, single PRP injections have no better effect than multiple PRP injections. But multiple PRP injections have a higher reduction in the intensity of pain when compared to single PRP injections during the follow-up period, according to the VAS score. According to the WOMAC score, there is no statistically significant difference in the treatment response with PRP injection between S-PRP and M-PRP groups. Further studies are needed with larger samples to understand the mechanism of PRP action in reducing pain and to evaluate if there is only a temporary symptom improvement or if PRP plays a more important role through disease-modifying properties.
